# Biosynfoni: a biosynthesis-informed and interpretable lightweight molecular fingerprint

**DOI:** 10.1186/s13321-025-01081-6

**Published:** 2025-08-29

**Authors:** Lucina-May Nollen, David Meijer, Maria Sorokina, Justin J. J. van der Hooft

**Affiliations:** 1 Bioinformatics Group, Wageningen University & Research, Droevendaalsesteeg 4, 6708 PB Wageningen, the Netherlands; 2 Present Address: Leiden Academic Centre of Drug Research, Leiden University, Wassenaarseweg 76, 2333 AL Leiden, the Netherlands; 3 DS&AI, Bayer Pharmaceuticals, Mullerstrasse, 178, 13353 Berlin, Germany; 4 Department of Biochemistry, University of Johannesburg, Street, Johannesburg, 2006 South Africa

**Keywords:** Natural products, Cheminformatics, Molecular fingerprint, Biosynthesis, Classification, Metabolomics, Biosynthetic building blocks, Metabolic modularity

## Abstract

Natural products provide a rich source of bioactive molecules for a variety of applications. Molecular fingerprints are the tool of choice for systematic large-scale studies of their structures. However, current molecular fingerprints insufficiently represent characteristic features of natural products inherently, decreasing the interpretability of natural product-specific predictions. Here, we show that a natural product-specific molecular fingerprint based on a relatively small set of selected biosynthetic building blocks provides more interpretable predictions of biosynthetic distance and natural product classification. Our fingerprint Biosynfoni outperforms MACCS, Morgan, and Daylight-like fingerprints in biosynthetic distance estimation, using 39 substructure keys. Moreover, Biosynfoni’s design, compactness, and concrete substructure definition allow easy visualisation of the detected substructures and their respective biosynthetic pathway origins. Through Biosynfoni, users can gain more insights from predictions and better examine the importance of features within machine learning models. Our results show that a short fingerprint consisting of biologically significant building blocks performs on par with top-performing molecular fingerprints for natural product classification while improving prediction explainability.

## Scientific contribution

Biosynfoni contributes to more interpretable and lightweight classification and retrobiosynthesis models by reflecting natural product biosynthetic information compactly and explicitly.

## Introduction

Natural products provide a rich resource of bioactive molecules [1, 2]. Their specialised nature provides evolutionarily optimised properties [3–5] useful in, among other fields [6, 7], pharmaceutics [8–12] and agriculture [13, 14]. The number of known natural product structures has been increasing with the advent of high-throughput analysis methods, currently comprising around 700,000 known natural products [15, 16]. Molecular structure descriptors provide a systematic way of storing, accessing, and analysing structural information of these natural product molecules. One type of descriptor, the molecular fingerprint, algorithmically describes a molecule’s substructures in a constant-length array. This constant length standardises input sizes, enabling straightforward comparisons between fingerprints and making them compatible with machine learning methods that require tabular data formats [17].

Molecular fingerprints are widely used for predicting quantitative structure-activity relationships (QSAR) but in their current form, they often lack interpretability [18]. Moreover, within natural product research, molecular fingerprints are not only used for activity prediction and structural comparisons, but also predictions of biosynthesis [19, 20, 21], classification [22], and taxonomic origin [23]. As such, they are a key asset in integrating structural information into bioinformatics workflows, leveraging implicit information about a compound’s origin, production and context [24–27]. Fingerprints for such applications ideally contain structural features that can be directly linked to the biosynthesis of the molecule. While many machine learning tools have been developed for natural product-related predictions [22, 23], the descriptors used as input often disregard differences in featurisation needs between drug-focused and natural product-specific applications. This consequently decreases the feature-based interpretability of the model output.

Here, we introduce the *Biosynfoni* fingerprint (from BIOSYNFOrmatic fiNgerprInt), which uses 39 structural features aligned with the biosynthetic nature of compounds, moving beyond purely structural characteristics. We achieve this through a substructure key-based fingerprint using building blocks and patterns described in Dewick’s 2009 book on natural product biosynthetic logic (Online Figures A25 and A26) [28]. This selection of building blocks and detection principles helps our fingerprint, Biosynfoni, to explicitly cover biosynthetically relevant structural features. We show that in comparison to other widely-used fingerprints, Biosynfoni better reflects biosynthetic distance, defined as the number of enzymatic steps separating two compounds in an enzymatic pathway, and performs on par in natural product-specific applications while providing enhanced explainability.

## Results

Biosynfoni’s fingerprint contains the number of substructure matches returned for each of the 39 substructure keys. We investigated Biosynfoni’s applicability domain and its performance in biosynthetic similarity representation, chemical space visualisation, and natural product classification.

### Biosynfoni’s 39 substructure keys capture the natural product chemical space

**Fig. 1:**
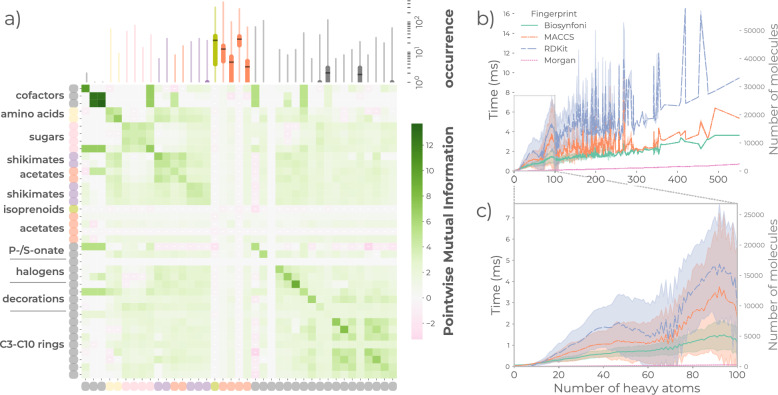
Applicability domain of Biosynfoni. **a** Occurrences (top) and co-occurrences (pointwise mutual information, bottom) of Biosynfoni substructure keys. Substructure keys are summarised by their larger group names and coloured by pathway, where grey symbolises lack of pathway. Occurrences are shown as bottomless box plots due to the logarithmic scale of the y-axis. Full labels are shown in Online Figure A24. **b**, **c** Generation times per number of heavy atoms shown for the entire set (b), and zoomed in for the most frequent sizes of 0–100 (c). Coloured areas represent standard deviation. The grey histogram represents the number of compounds in the dataset per heavy atom number

We investigated Biosynfoni’s applicability domain in natural products by assessing structure coverage, substructure occurrence, and relative fingerprint generation times for the entire COCONUT natural product dataset. The average atom coverage of 97% indicates Biosynfoni captures almost all of the atoms in the natural products’ structures at least once with its 39 substructure keys (Online Figures A1 and A2). The average counts and count variation differ between substructure keys, with acetate pathway substructure keys showing the highest average counts and count variations (Fig. 1a, top). Most other substructure key types have an average count between one and zero, except for hydroxyl groups and 6-carbon rings. Fluorine substructures had zero counts over the entire dataset, and inspection of the dataset SMILES confirmed the absence of fluorine in the dataset used. The high average counts for the acetate pathway substructure keys are due to their small and simple carbon structure that can be detected in multiples throughout all-natural products. Several substructure keys frequently co-occur (Fig. 1a), especially substructure keys within the same pathway class, as shown by the higher pointwise mutual information scores for same-pathway substructure keys (e.g., co-occurrence of sugar moieties). Natural products of particular classes can indeed consist of multiple building blocks originating from the same biosynthetic basal pathway. For instance, terpenoids tend to contain multiple isoprene units, while polyketides tend to contain several acetate pathway building blocks [28, 29].

Besides chemical coverage, we also tested the computational performance of Biosynfoni. The fingerprint generation (Fig. 1b, c) for Biosynfoni is faster than both MACCS and RDKit fingerprint generation, despite Biosynfoni’s pure-Python coding, but slower than Morgan fingerprint generation. We also note that with increasing compound size, the generation time also increases less steeply than the MACCS and RDKit fingerprints.

### Biosynfoni captures biosynthetic changes along biosynthetic reaction chains

**Fig. 2:**
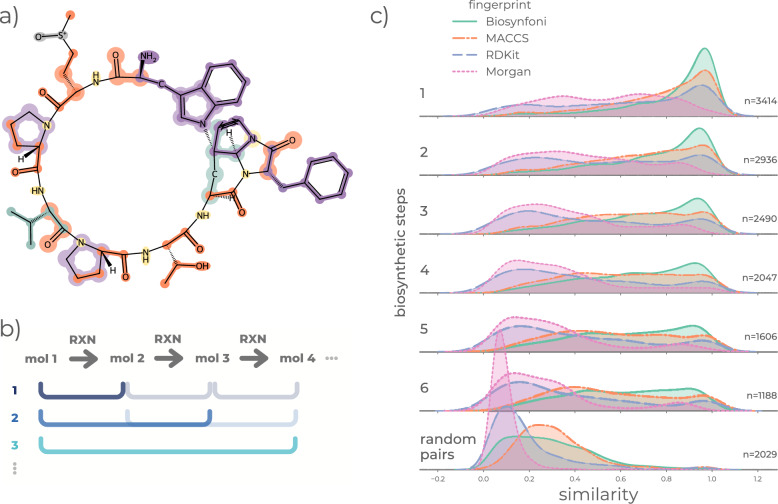
Biosynfoni as a biosynthetic information-aware fingerprint. **a** Illustration of the core idea behind Biosynfoni. For a given natural product compound, Biosynfoni detects the compound’s constituent building blocks. The most important building blocks have a corresponding biosynthetic origin pathway, as shown by the corresponding colour of the building block. Biosynfoni detects building blocks in an overlapping fashion, as partially illustrated by some thicker, lighter-coloured highlights. For example, in the left bottom of the molecule, an acetaldehyde substructure (thicker light-orange highlight) overlaps with isoprene one (thinner teal highlight). **b** Compound pairs with increasing biosynthetic distance. For one biosynthetic reaction chain, compound pairs of increasing biosynthetic distance are obtained, and similarity is calculated to obtain (c). **c** Fingerprint similarity as a proxy for biosynthetic similarity per fingerprint. As the biosynthetic distance increases (increasing numbers of biosynthetic reactions on the left), the similarity decreases. This is most gradually and clearly visible for Biosynfoni. Biosynthetically related compounds also show higher similarity compared to non-related compounds (control), with the distinction being clearest in Biosynfoni. The sample size per step is indicated by *n*

In natural products, our Biosynfoni fingerprint captures biosynthesis building blocks like amino acids and isoprene units that can overlap with each other (Fig. 2a). To investigate if Biosynfoni is able to more accurately capture biosynthetic similarity than competitive fingerprints, we calculated Tanimoto similarity scores for compounds from biosynthetic reaction chains, for pairs with increasing numbers of metabolic reaction steps between them (Fig. 2b). We compared these to the Tanimoto similarities derived from other fingerprints (Fig. 2c). For compound pairs with few biosynthetic reaction steps between them, Biosynfoni similarity scores are largely higher than for other fingerprints. Unlike the other fingerprints tested, Biosynfoni shows a continuous decrease in similarity scores as more reactions separate compound pairs. Simultaneously, random pairs have low similarities, comparable to the random-pair similarity scores of the other fingerprints. Thus, Biosynfoni is able to better resolve biosynthetically closely related, less related, and unrelated pairs. Remarkably, it captures biosynthetic changes with 39 substructure keys, despite the fact that compound structure is a lossy representation of biosynthesis, with metabolic transformations not always being directly visible and recognizable in the final structure of the compound.

### Biosynfoni can be used for chemical space visualisation and interpretable natural product class prediction

**Fig. 3:**
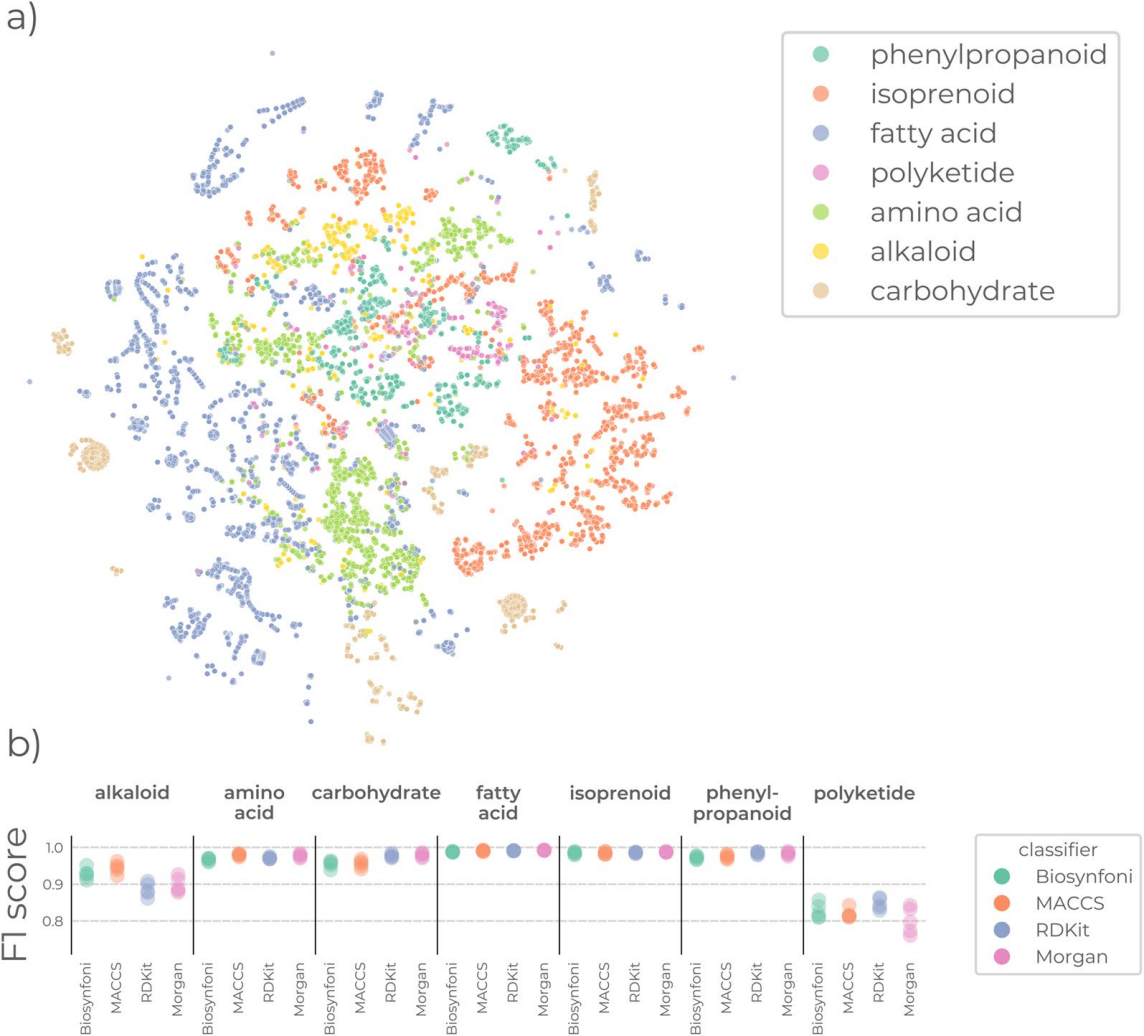
Biosynfoni applications **a** t-SNE of Biosynfoni-distances of ChEBI compounds, coloured by annotated class. For visualisation purposes, compounds belonging to multiple classes were left out. **b** F1-scores per fingerprint over 5-fold cross-validation test sets for a Random Forest model for natural product class prediction. Other metrics are available in Online Figure A16, and comparative performances of training and test sets are available in Online Figure A17

Biosynfoni can also be used like other fingerprints to visualise chemical space. We used t-SNE [30] and UMAP [31] dimensionality reduction methods on the Biosynfoni-based similarity matrix to visualise the chemical space of compounds as captured by Biosynfoni (Fig. 3a and Online Figures A12, A13, A14 and A15). The t-SNE shows distinct areas occupied by certain natural product classes, indicating fingerprints are more similar for the compounds of those classes (Fig. 3a).

**Fig. 4:**
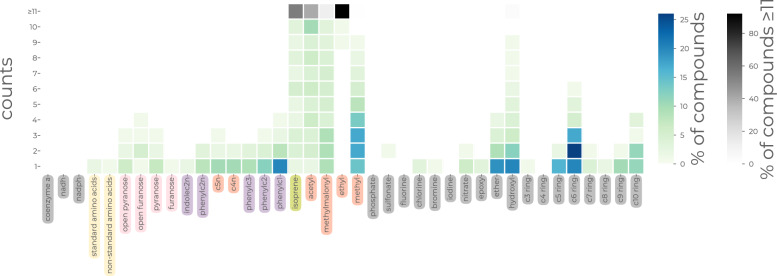
QR code visualisation of Biosynfoni fingerprints of all natural products of the COCONUT database. The x-axis shows the substructure keys of Biosynfoni, while the y-axis shows the number of times the substructure occurs within one molecule. The colour intensity shows the relative number of molecules with each count-value, e.g. 20% of natural products have one hydroxyl group, 5–10% have two hydroxyl groups (third peak from the right, bottom-most square). All substructure count numbers above 11 are grouped together for visualisation in the top row, with a separate colour scale to maintain distinguishability for the other values

Additionally, Biosynfoni can be used to visualise molecular datasets and differences between datasets in a completely new way. Figures 4, and Online Figures A3, A4, A5, A6, A7, A8, A9, A10 and A11 show heatmaps of Biosynfoni substructures and their counts for a dataset, coloured increasingly darker as more molecules display that count. If traditional fingerprints are molecule ‘barcodes’, Biosynfoni now allows molecule QR codes and average QR codes over datasets (Fig. 4). These QR plots allow for an instant grasp of the chemical space of a molecular set. We can instantly see if the fingerprint distributions differ between different classes or sets, which is the case for ChEBI’s classes and natural and synthetic compounds (Online Figures A3, A4, A5, A6, A7, A8, A9, A10 and A11).

To assess natural product class prediction, we trained Random Forest multilabel classifiers on the ChEBI-annotated labels and assessed performance using 5-fold cross-validation. For machine learning model training, it is advantageous to use fewer predictors, as this reduces the time and memory needed in the training process. Indeed, Biosynfoni-based model training had the lowest peak memory usage, and Biosynfoni and MACCS had the lowest training times of 17.4 and 17.6 s for a full model. In comparison, the significantly larger Morgan and RDKit fingerprint took 55.6 and 61.1 s, respectively, a roughly threefold difference (Online Figure A18). Despite the lower number of predictors, the Biosynfoni-based classifier showed similar classification performance to the other fingerprint-based classifiers (Fig. 3b and Online Figure A16). Model overfitting levels were comparable across fingerprints (Online Figure A17). Moreover, the Biosynfoni-based classification decisions can be easily interpreted due to the concrete and biologically relevant substructure keys. For example, in the multilabel classification of natural product classes, the isoprene substructure key has the highest feature importance (Online Figure A19), likely because it is important in differentiating terpenoids and other honeycomb-shaped molecules. While the classification performance can be further improved through optimisation of model architectures, hyperparameter optimisation, and more equal class distribution of the multi-class training data, these results serve as a proof of concept of Biosynfoni’s applications in natural product research.

Biosynfoni improves interpretability in such applications because its values directly represent substructures (as opposed to hashed fingerprints), and those substructures are biosynthetically meaningful (as opposed to other substructure key fingerprints). The smaller fingerprint length allows for clearer visualisations compared to longer fingerprints, as shown in the comparison between Biosynfoni (Online Figure A19) and MACCS feature importances (Online Figure A20). Moreover, the fact that the fingerprint is counted, and the further categorisation of the substructure keys into their main biosynthetic pathway origins (Online Figure A26) allows for new chemical space visualisations (Fig. 4). Our Biosynfoni natural product classification tool (Online Figure A29) illustrates possibilities for improved visualisation and interpretation of model predictions, highlighting which biosynthetic pathway building blocks contribute to the classification outcome.

## Conclusion

Biosynfoni is a compact substructure-based molecular fingerprint providing increased interpretability for various natural product cheminformatic applications. With its small set of 39 selected substructure keys, it performed similarly to larger MACCS, Morgan, and RDKit fingerprints in natural product class prediction tasks. Additionally, its small size allows faster and lower memory model training. Its biosynthetically relevant substructure keys and its counted nature offer enhanced biosynthetic information capture and interpretability, further enhanced through the molecular QR codes introduced here. This biochemical interpretability helps to gain trust in prediction results for experimental workflows, while the flexible implementation allows for the addition and removal of substructure keys to customise for extended or other applications.

## Methods

### Materials

#### Tools

We used Python [32] (v3.13.1) and the cheminformatic RDKit [21] package (v2024.09.4) as our main tools in Biosynfoni’s creation and testing. Table A2 and A3 contain a detailed list of other tools and materials used, and their respective versions.

#### Compound structures and metadata

We used the second version (September 2024) of the COCONUT database [15, 33] as our primary natural product structure source. Any compounds from the .sdf+ file that could not be read by RDKit were left out, leaving us with 695,129 compounds.

As the COCONUT compounds were only annotated with predictions of their natural product class, rather than manual annotations, we needed to obtain a ground truth dataset for our classification tasks. For this, we used ChEBI’s [34] three-star database (v. 2023.10.27). We extracted the class annotations from the ChEBI ontology .owl file (Table A3)) using Protégé [35]. We matched the three-star database compounds to the extracted classifications using their compound IDs, resulting in 16,828 compound-label pairs. Supplementary material Table A1 shows the number of compounds per class, where we note that compounds can have multiple class labels.

To assess biosynthetic similarity, we first obtained reaction chains from MetaCyc [36] pathways. We filtered for pathways that included at least one natural product and were at least six reactions long. Natural products were identified through InChI comparison to COCONUT compounds, where InChI parts 1–3 are identical (i.e., the compounds possess identical atom connectivity). For each pathway, we extracted the longest chain of consecutive reactions, resulting in 486 reaction chains. From each chain, we extracted ((*x*, $x+1$), (*x*, $x+2$),..., (*x*,$x+n$)) reaction pairs, where *n* represents the number of reactions between two compounds (Fig. 2b).

To visualise the differences in Biosynfoni fingerprints between natural products and synthetic compounds, we used the non-biogenic ZINC database subset (3,019,372 compounds) as shared by [37].

### Methods

#### Substructure key selection

For the fingerprint substructure keys, we selected a representative set of core structure building blocks and informative decorative structures, based on Dewick’s 2009 book on natural product biosynthesis [28]. We included his set of eight fundamental biosynthetic building blocks, each of them linked to a main metabolic pathway branching out from glycolysis (Online Figure A25). To these, we added amino acids and sugars, as amino acids are of special biochemical interest regardless of their individual precursor pathways [38], and sugar moieties are an often-occurring modular addition upstream of glycolysis [29]. We then added decoration substructures like hydroxyl groups, rarer-element decorations, special-sized carbon rings, and helper molecules such as coenzymes. In total, this added up to 39 substructure keys (Online Figure A26).

#### Biosynfoni creation

To implement the fingerprint, we used RDKit’s Chem.Mol.GetSubstructMatches function for substructure key detection. Substructure keys are defined in SMILES ARbitrary Target Specification (SMARTS) to allow for regular expression-like detection. The substructure definitions maintain strict atom identity (i.e., element) requirements but allow complete flexibility in bond order (i.e., either single, double, aromatic, etc.). We chose this representation, because in biosynthesis any atom has to originate from a specific source, and thus atom identity encodes biosynthetic origin, while bonds change with reactions and thus encode less biosynthetic origin information. We also note that substructure detections can overlap (see Fig. 2a). To ensure transparency and interpretability, further explanations of SMARTS representations can be found in the substructureSmarts dictionary in Biosynfoni’s subkeys module. We note that we did not include chirality in the substructure keys as most current molecular databases have incomplete or inaccurate chirality information [33]. Future Biosynfoni iterations can explore chirality when chirality-complete databases show chirality influences in detection fuzziness and subsequent distance changes of chirality-altering biosynthetic reactions. We note that the Biosynfoni implementation allows adjustment of the substructure keys for any specific chirality-inclusion.

We labelled each of the Dewick substructures with their corresponding metabolic pathways, shikimate, acetyl, terpenoid, carbohydrate, and amino acid (Online Figure A25). Amino acids have their own annotation regardless of their relation to up- or downstream pathways, due to their interwovenness into various metabolic pathways, distinct role as a primary metabolic building block, and consequent difficulty of traditional pathway assignment. These labels serve mostly as an interpretability aid for feature importances of Biosynfoni-based prediction models. Decoration structures do not have a specific pathway annotation.

#### Time performance and structural coverage

To compare time performance, we measured generation times of Morgan [39], (Daylight-like) RDKit [21], MACCS [40], and Biosynfoni fingerprints over all COCONUT compounds. We used radius 2 and non-chirality for Morgan fingerprints and standard parameters for everything else, resulting in 2048-bit Morgan and RDKit fingerprints and 167-bit MACCS fingerprints. Generation time was measured on a 2021 14" MacBook Pro with an M1 Pro chip and 16GB RAM. To assess how universally Biosynfoni can capture natural product structure, we measured Biosynfoni’s atomic coverage on COCONUT compounds by counting substructure-detected heavy atoms divided over all heavy atoms.

#### Biosynthetic similarity estimation

For biosynthetic similarity assessment, we took the MetaCyc reaction chain pairs as described in 5.1.2. We generated Morgan, RDKit, MACCS, and Biosynfoni fingerprints for all molecules included in the MetaCyc reaction chain pairs dataset. For each molecule pair (*x*, $x+n$) with *n* ($n \le 6$) reactions between them, we computed Tanimoto similarity between the two compounds for each fingerprint type. Randomly shuffled MetaCyc molecule pairs from the same set served as a control for each fingerprint’s ‘baseline’ similarity. We visualised the similarity scores per *n* through kernel density estimate plots of the similarities (Fig. 2c). To additionally visualise individual values per fingerprint, we plotted similarities for each molecule pair in scatter plots (Online Figures A21, A22 and A23).

To calculate similarity we used the metric of choice in cheminformatics [41], the Tanimoto coefficient [42]. Because most Tanimoto similarity implementations treat arrays as binary rather than counted (Eq. 1), we implemented a counted Tanimoto similarity, also known as the multiset Tanimoto coefficient calculation as defined in Eq. 2.

1 $$\begin{aligned} T(A, B)= & \frac{|A \cap B|}{|A \cup B|} \end{aligned}$$

2 $$\begin{aligned} T(A, B)_{\text {multiset}}= & \frac{\sum _{i=1}^{n} \min (A_i, B_i)}{\sum _{i=1}^{n} \max (A_i, B_i)} \end{aligned}$$

#### Chemical space visualisation

To assess chemical space visualisation capabilities, we calculated similarity matrices of the compounds in the ChEBI dataset for the Morgan, RDKit, MACCS, and Biosynfoni fingerprints. We then visualised these distances through dimensionality reduction methods, colouring compounds by their ChEBI annotated class, using only compounds with a single assigned class. We used both t-SNE [30] (perplexity=50, n_iter=2000) and UMAP [31] for dimensionality reduction (default settings), as suggested in literature[43].

#### Classification performance

To see how Biosynfoni performs as featurisation for Machine Learning-based natural product classification, we trained a Random Forest classifier on the ChEBI dataset, with default settings, 10000 predictors, and a maximum depth of 100 splits. We compared classification performance with 5-fold cross-validation, obtaining performance statistics and feature importances for both training and test predictions.

## Additional file

Supplementary file 1 (pdf 5819 KB)

## Data Availability

Code was written in Python (v3.11.5). Source code and experimental scripts can be found at https://github.com/lucinamay/biosynfoni. Version 1.0.0 of Biosynfoni, including experimental scripts, was also archived on Zenodo at https://zenodo.org/records/14822624. Molecular structures of the biosynthetic reaction chains and extracted classification labels can be found on Zenodo at https://doi.org/10.5281/zenodo.14791205. The trained classifiers can be found at https://doi.org/10.5281/zenodo.14791239. Other data used for experiments are publicly available at the respective sources mentioned in Table A1 and Table A2. The natural product class-prediction tool based on the trained Biosynfoni-based classifier can be used at https://moltools.bioinformatics.nl/biosynfoni. More information on the Biosynfoni web application can be found in Online Figure A28.

## Abbreviations

InChI: International Chemical Identifier
QSAR: Quantitative structure-activity relationships
t-SNE: T-distributed stochastic neighbor embedding
UMAP: Uniform Manifold Approximation and Projection
SMILES: Simplified Molecular Input Line Entry System
SMARTS: SMILES ARbitrary Target Specification
