# Supplementary material for: Biosynfoni: a biosynthesis-informed and interpretable lightweight molecular fingerprint
Source: J Cheminform. 2025 Aug 29;17:136. doi: 10.1186/s13321-025-01081-6 (PMC12395878; doi:10.1186/s13321-025-01081-6)
Supplement: Supplementary file 1 — (pdf 5819 KB) [file 13321_2025_1081_MOESM1_ESM.pdf]

Appendix A Supplementary Materials

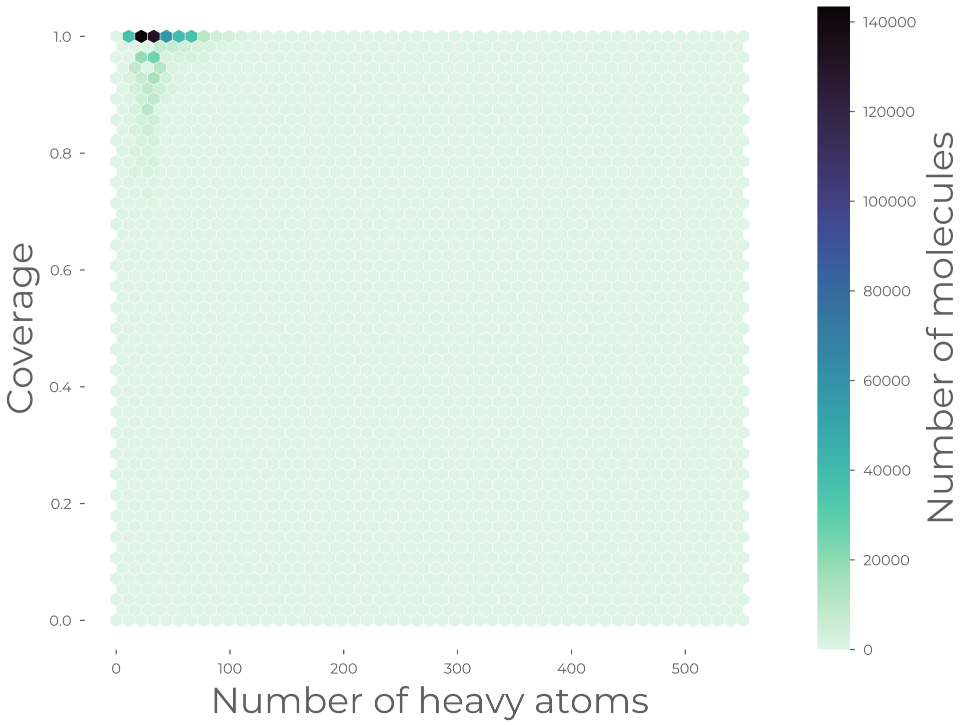

Fig. A1: Biosynfoni's atomic coverage of all COCONUT compounds. The number of heavy (non-H) atoms is used to indicate molecular size. Darkly coloured hexagon bins indicate many compounds with that given coverage.

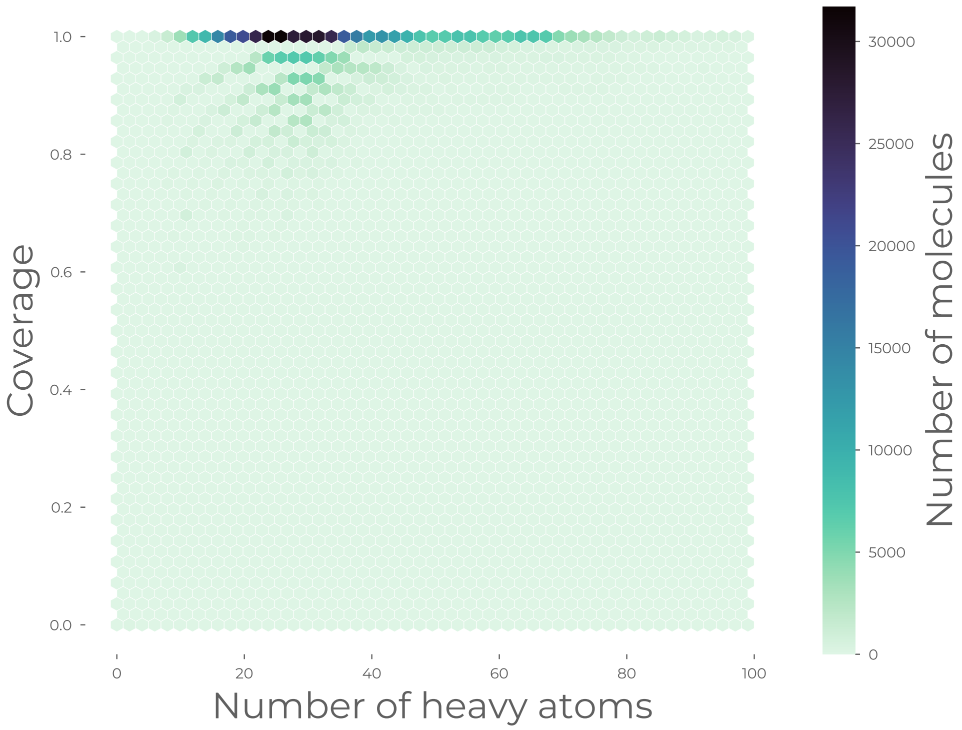

Fig. A2: Biosynfoni's atomic coverage of all COCONUT compounds with a heavy (non-H) atom count of ¡100..

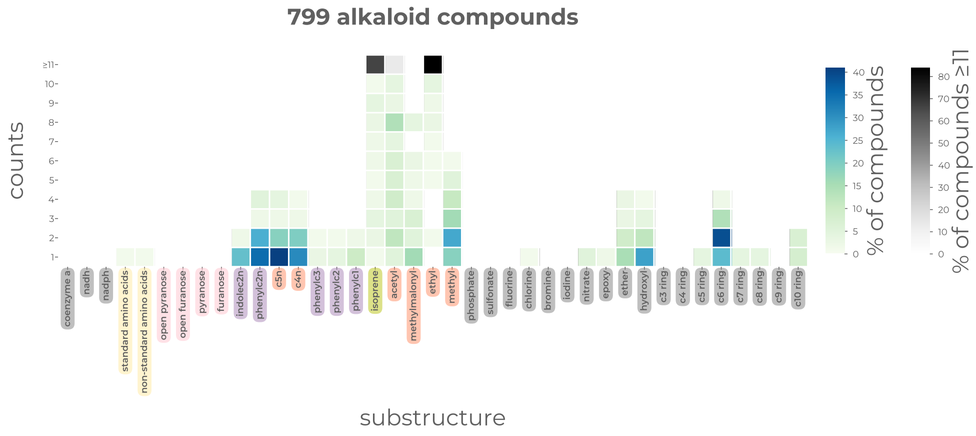

Fig. A3: QR of natural product compounds for various classes: Compounds only annotated as 'alkaloid'

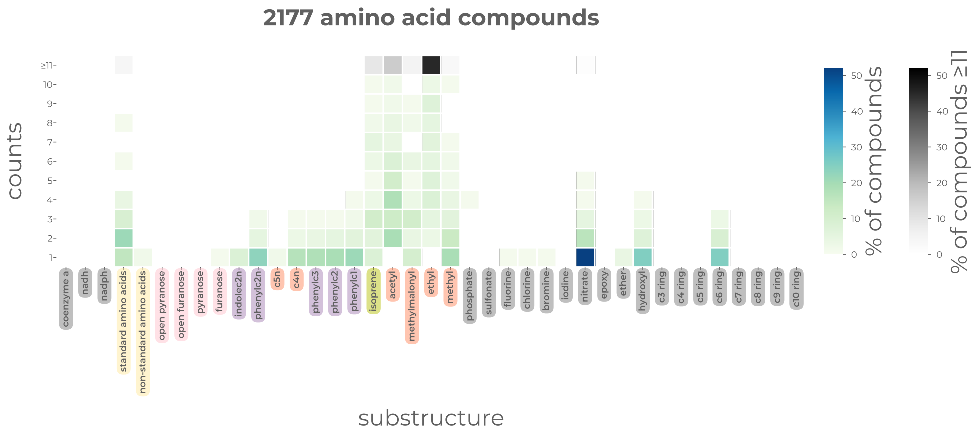

Fig. A4: Compounds only annotated as ’amino acid’

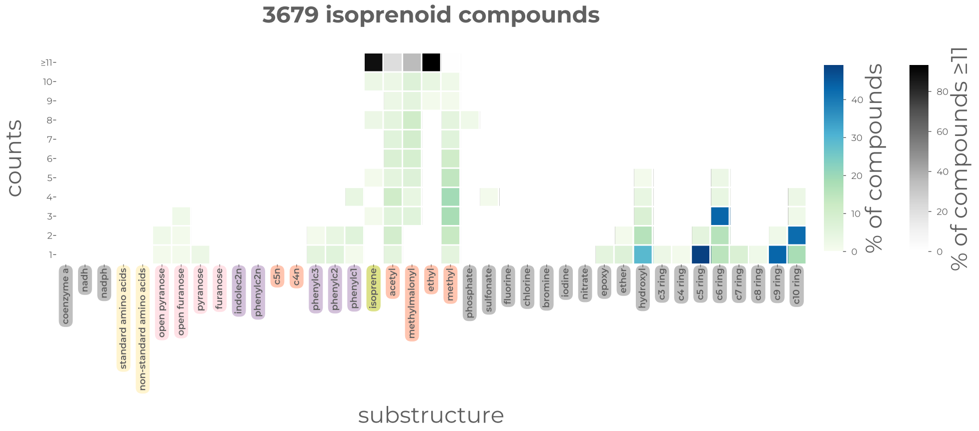

Fig. A5: Compounds only annotated as ’isoprenoid’

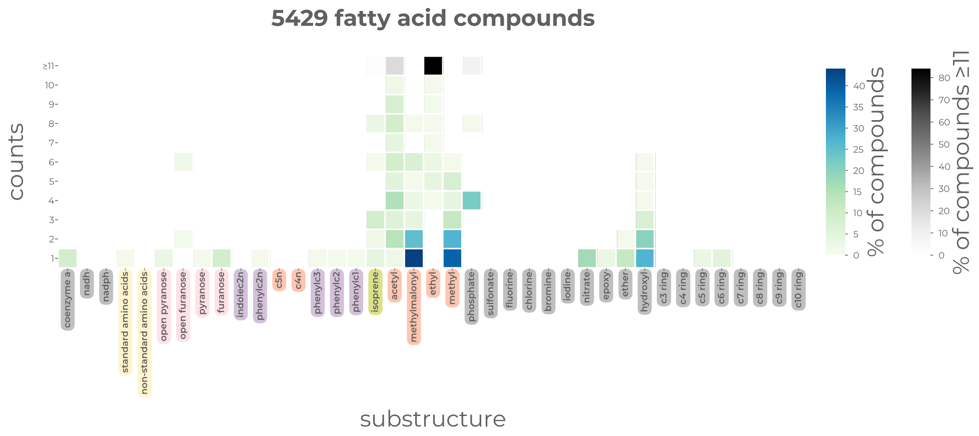

Fig. A6: Compounds only annotated as 'fatty acid'

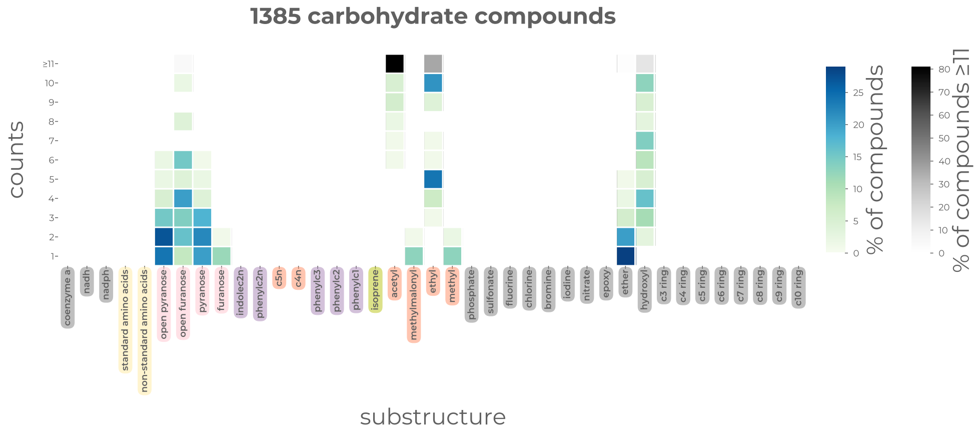

Fig. A7: Compounds only annotated as 'carbohydrate'

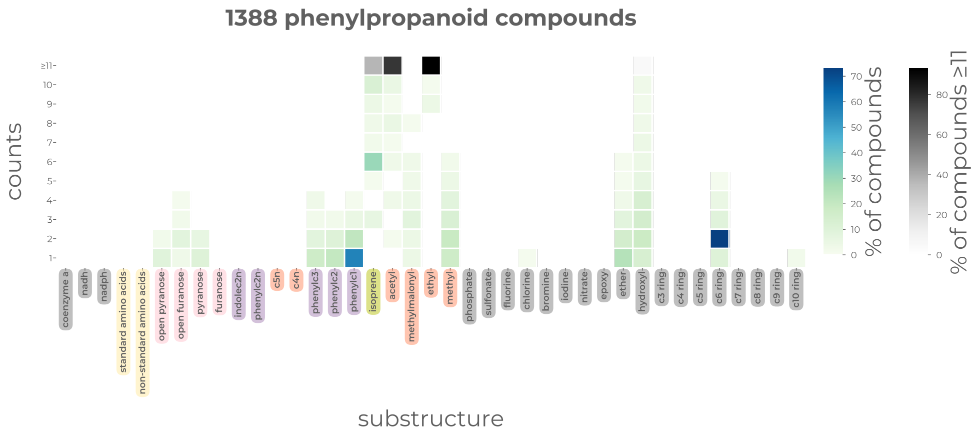

Fig. A8: Compounds only annotated as 'phenylpropanoid'

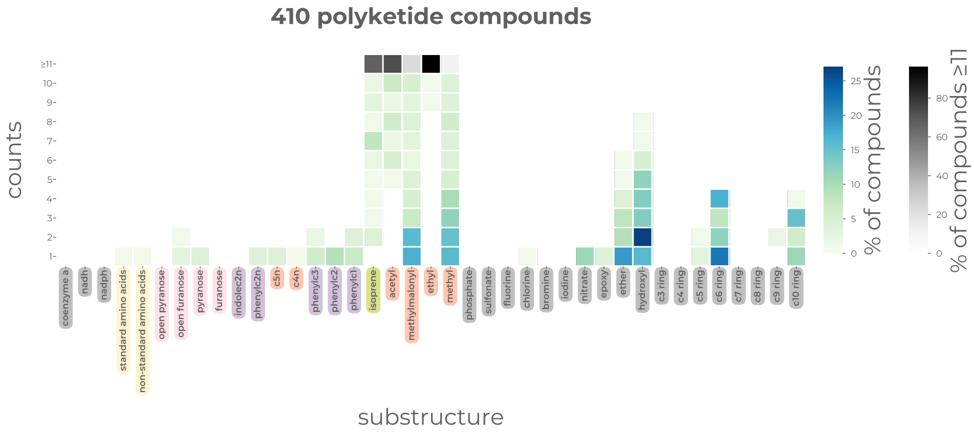

Fig. A9: Compounds only annotated as 'polyketide'

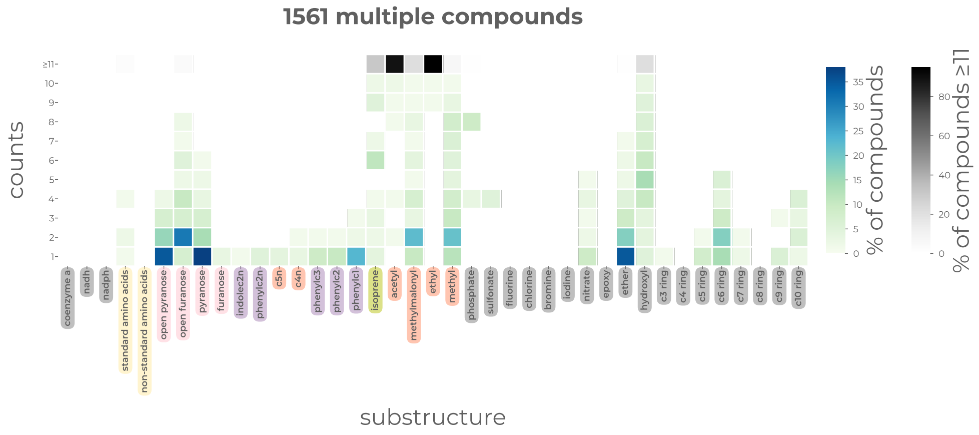

Fig. A10: Compounds annotated as multiple classes'

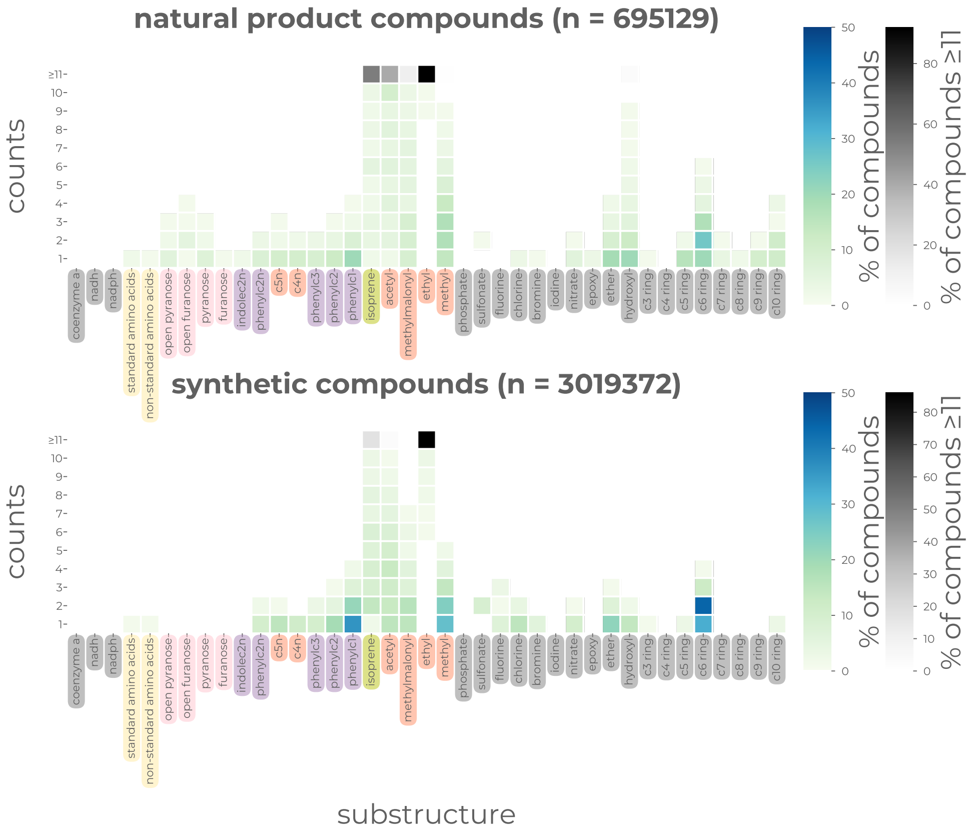

Fig. A11: QR visualisation of natural product vs synthetic compounds.

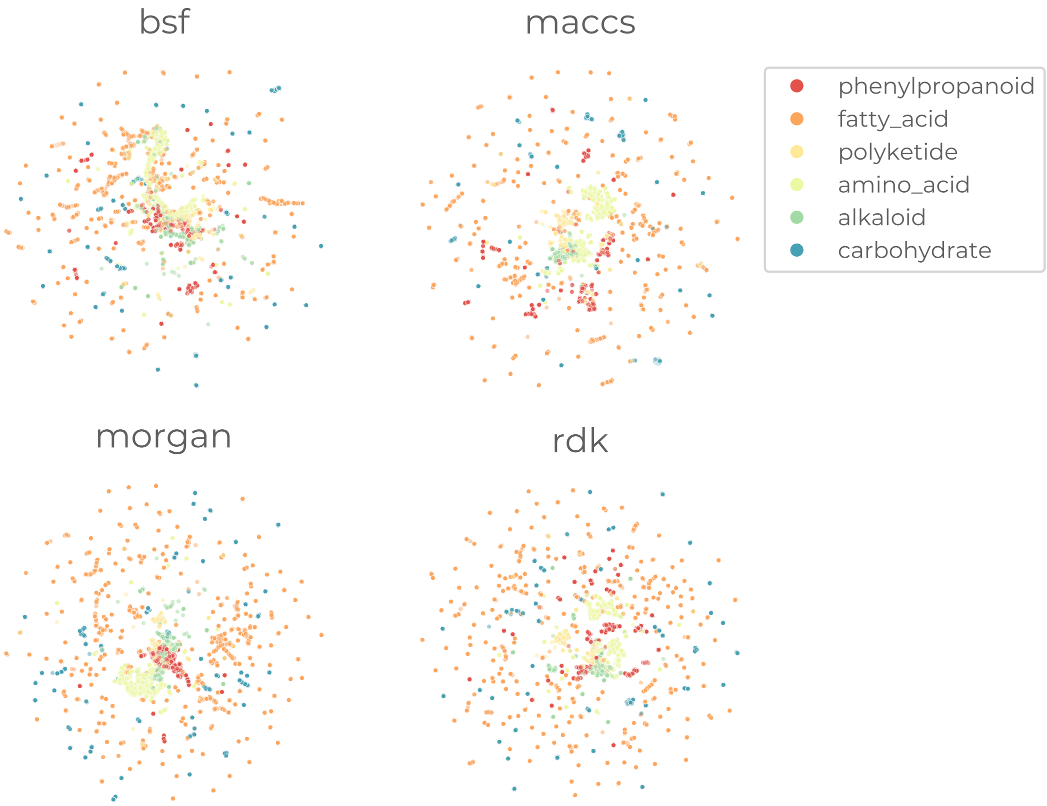

Fig. A12: UMAP visualisation of chemical space of different natural product classes (only compounds with a single class label). In ChEBI natural product class annotation, isoprenoids are always classified as both isoprenoid and fatty acid, so they all have multiple class labels.

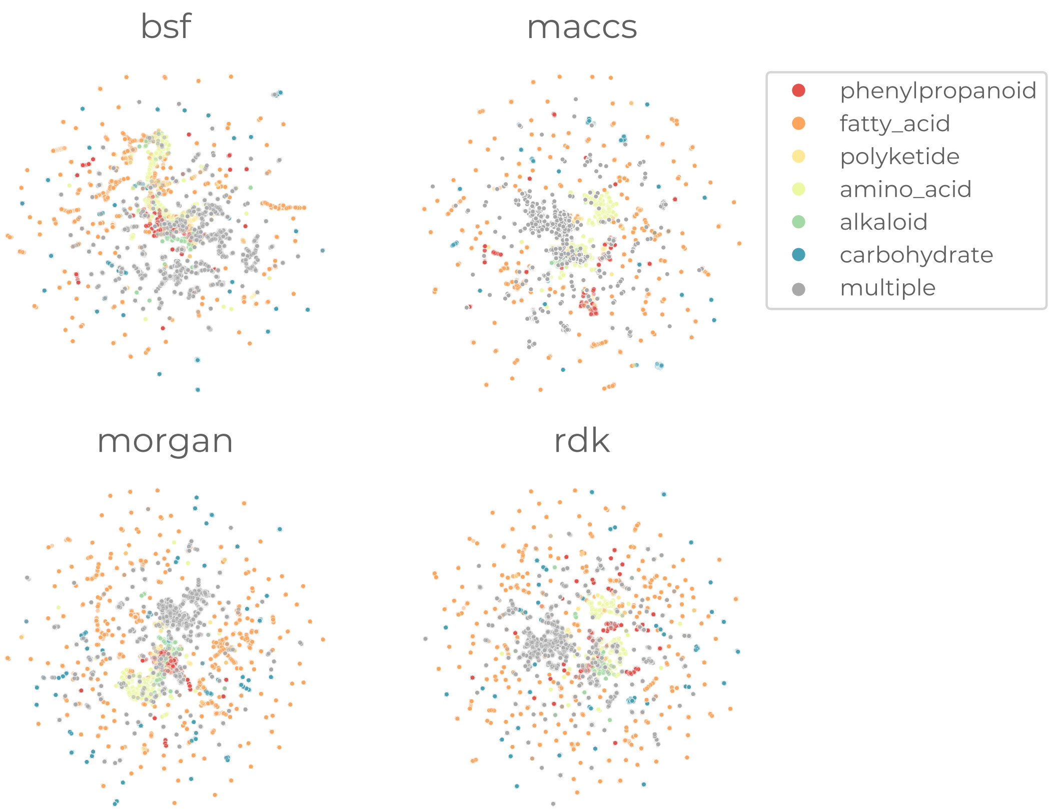

Fig. A13: UMAP visualisation of chemical space of different natural product classes (including compounds classified as several classes). In ChEBI natural product class annotation, isoprenoids are always classified as both isoprenoid and fatty acid, so they all have multiple class labels.

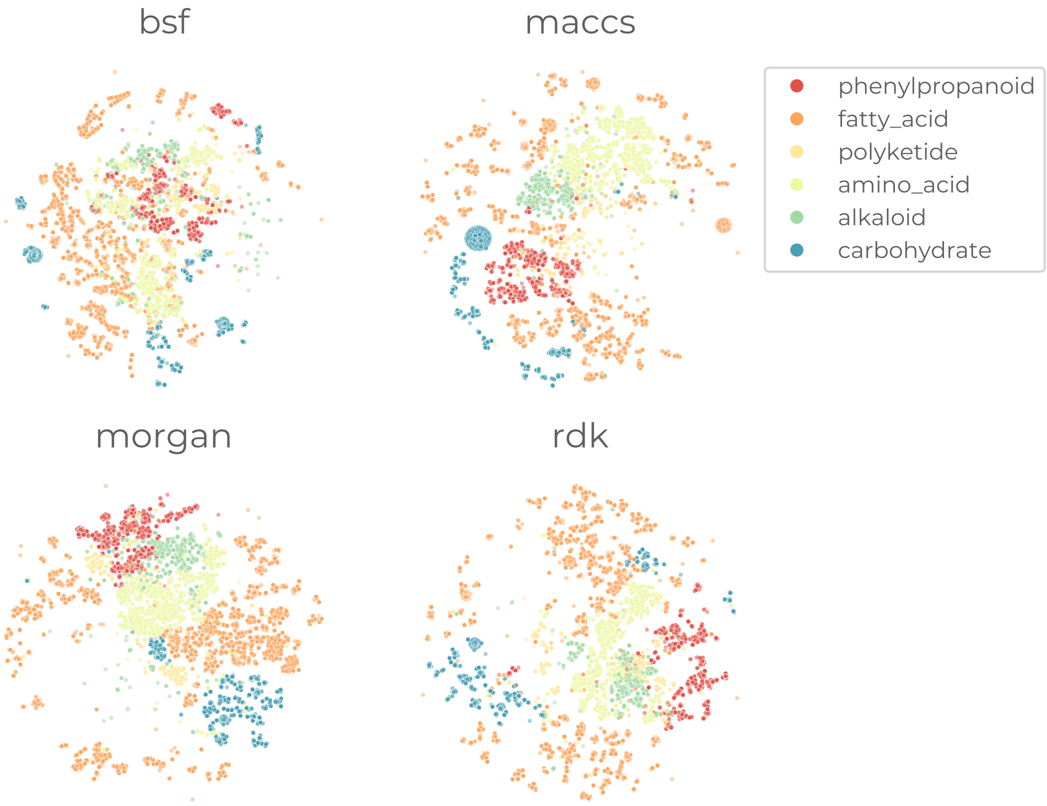

Fig. A14: t-SNE visualisation of chemical space of different natural product classes (only compounds with a single class label). In ChEBI natural product class annotation, isoprenoids are always classified as both isoprenoid and fatty acid, so they all have multiple class labels.

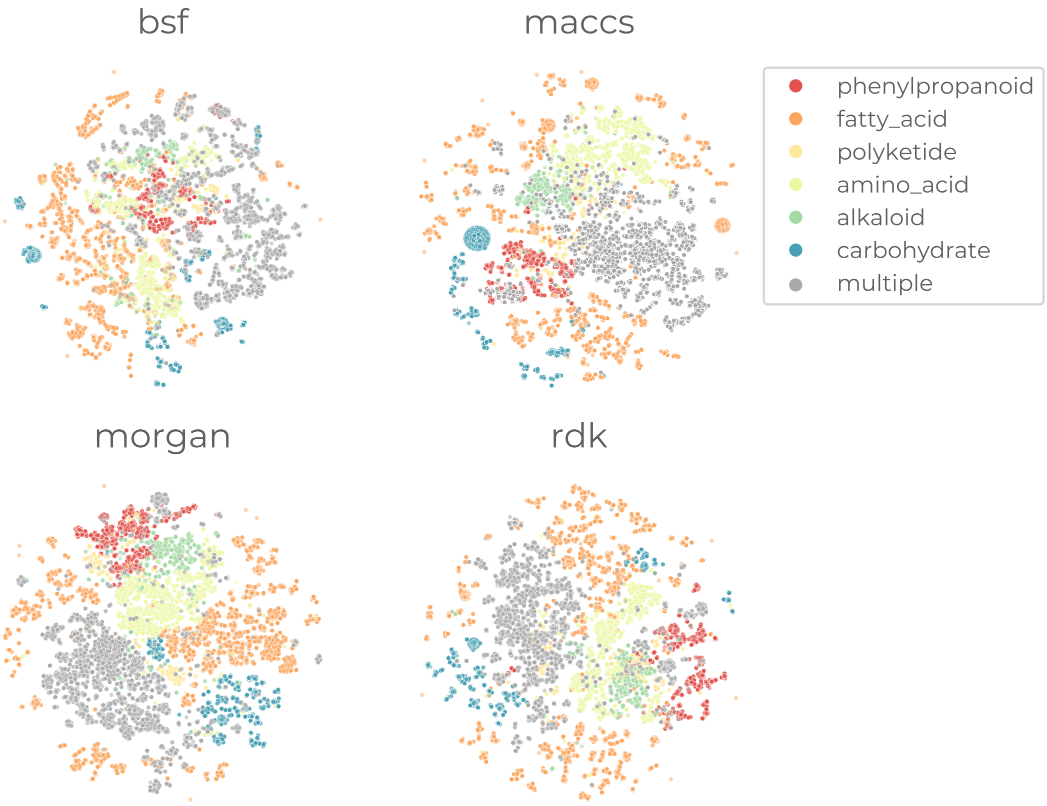

Fig. A15: t-SNE visualisation of chemical space of different natural product classes (including compounds classified as several classes). In ChEBI natural product class annotation, isoprenoids are always classified as both isoprenoid and fatty acid, so they all have multiple class labels.

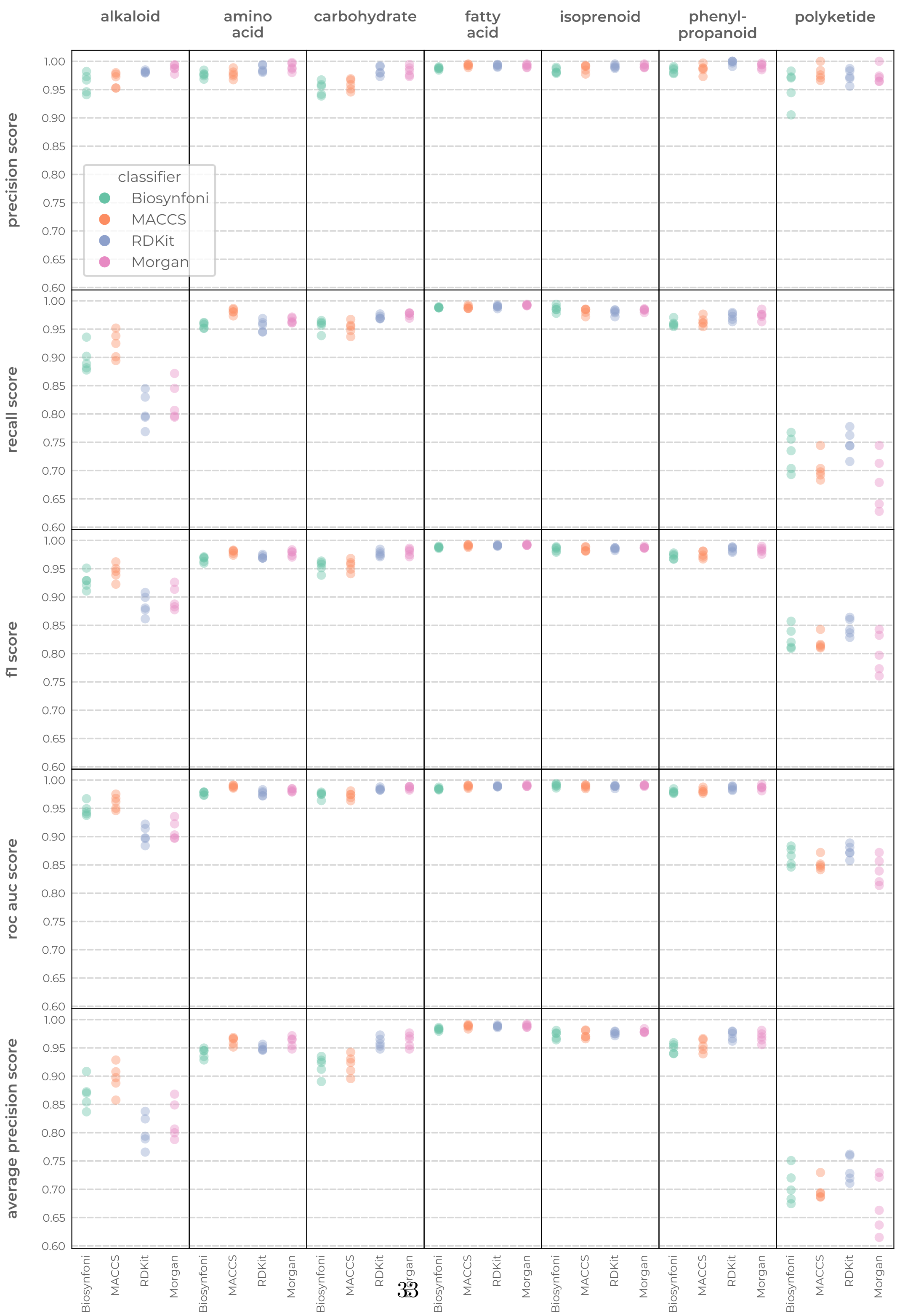

Fig. A16: Classification performance over 5-fold cross-validation of multilabel Random Forest models predicting natural product classes.

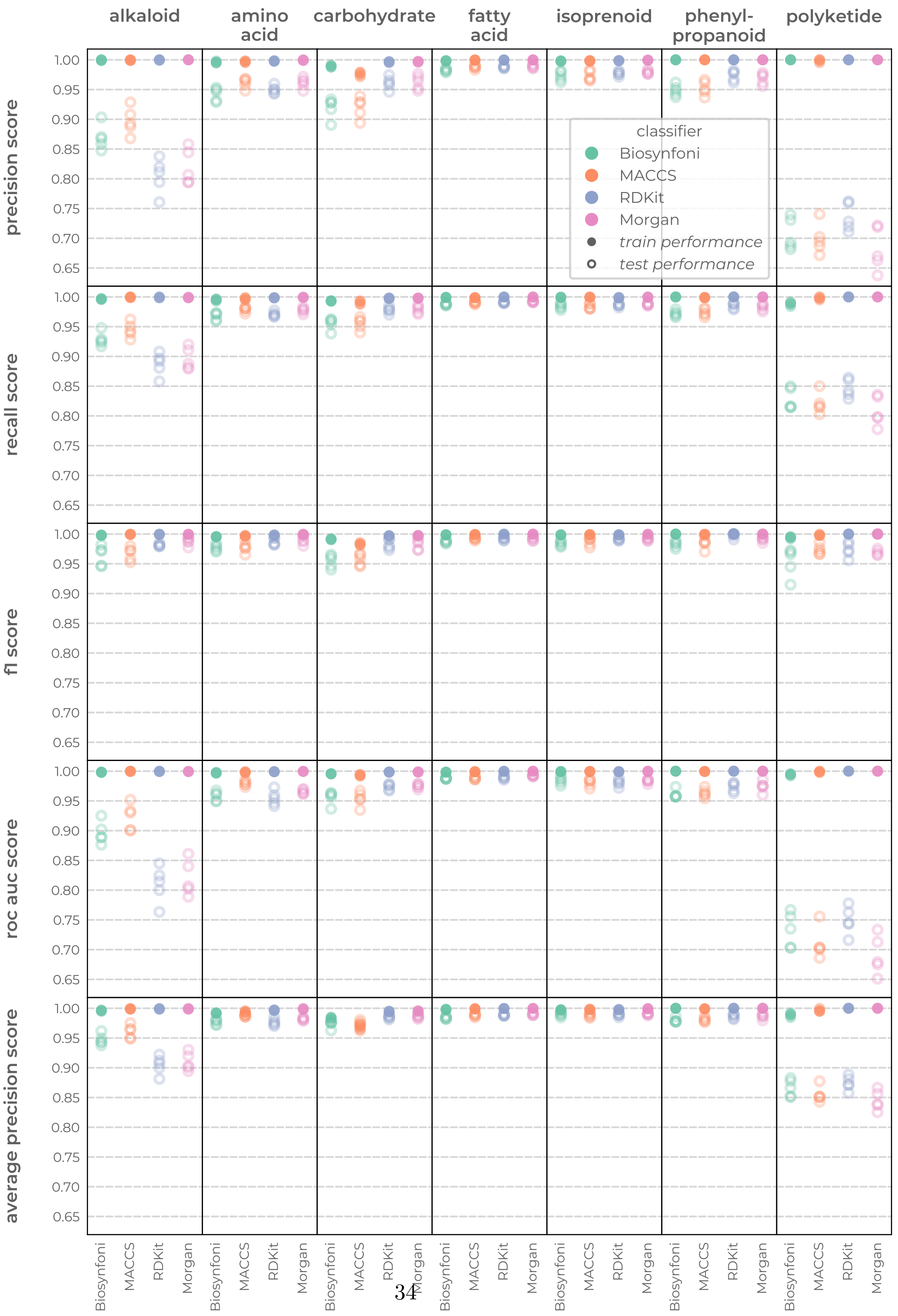

Fig. A17: Classification performance on training and test set over 5-fold cross-validation of Random Forest models predicting natural product classes. Similar reductions of training-to-test set prediction performance suggest overfitting levels of Biosynfoni-based models are similar to those of other fingerprint-based ones

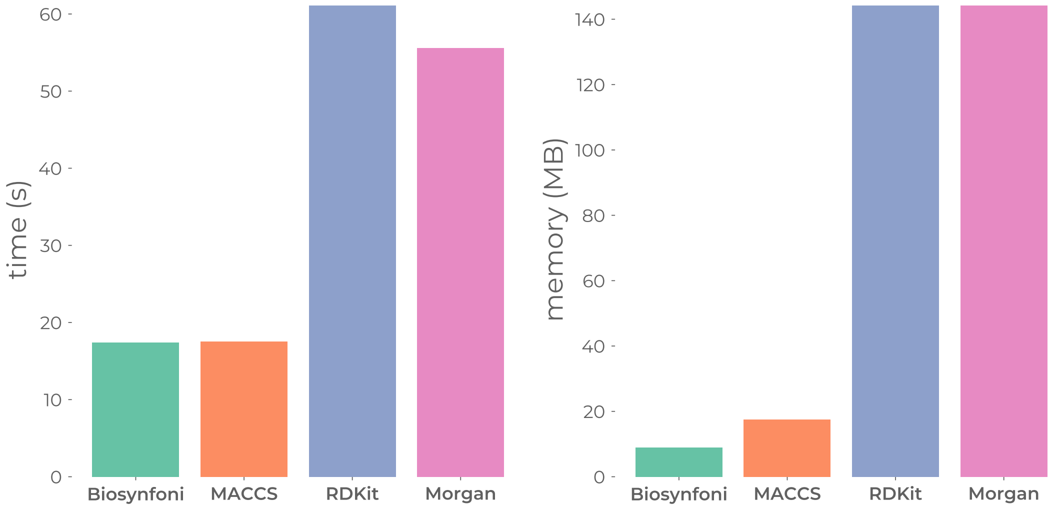

Fig. A18: Time and peak memory taken to train a Random Forest model on the full classification dataset

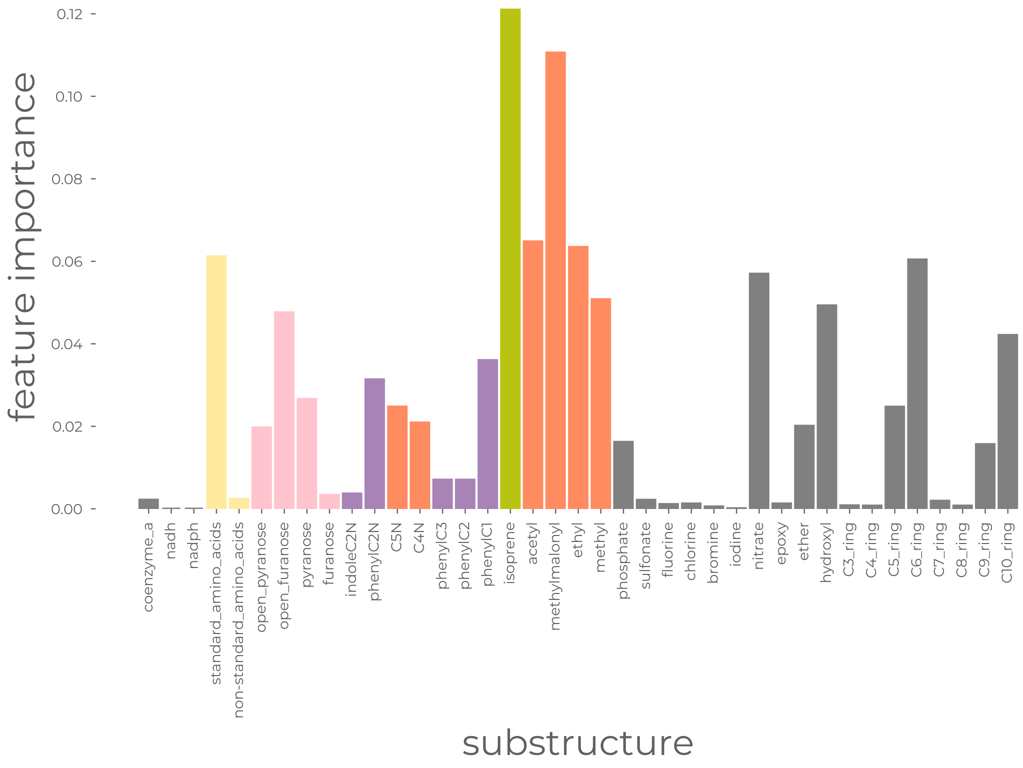

Fig. A19: Feature importances for the Biosynfoni Random Forest classifier trained on the full classification dataset. Building block parent pathways are indicated by colour (Figure A25), where grey is no assigned pathway.

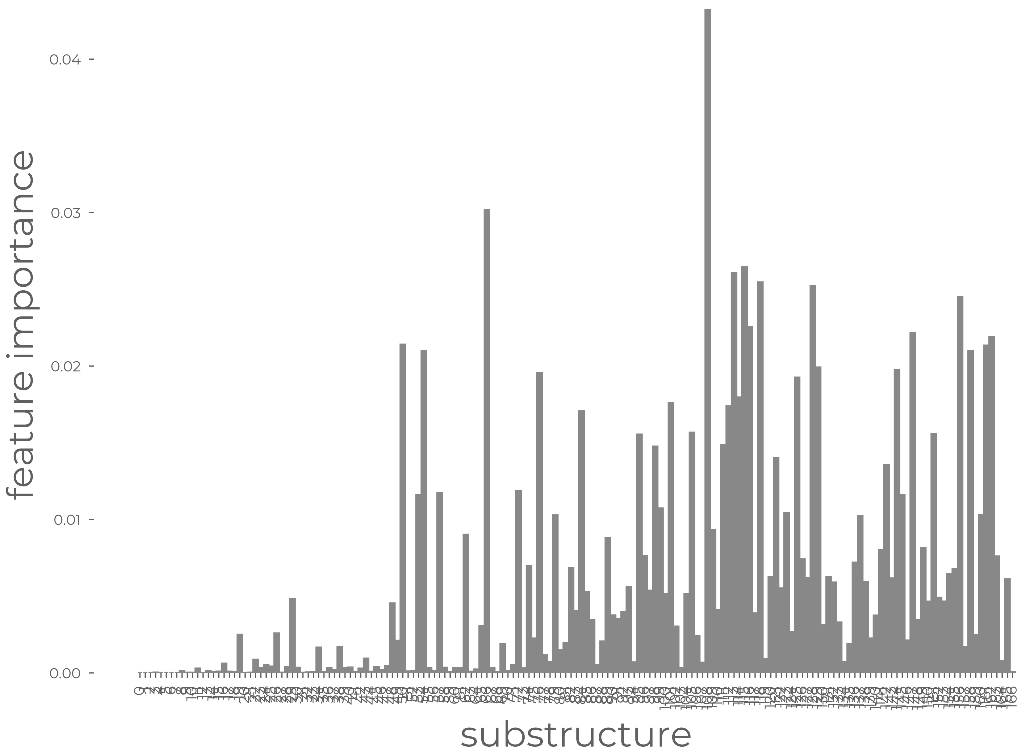

Fig. A20: Feature importances for the MACCS Random Forest classifier trained on the full ChEBI set.

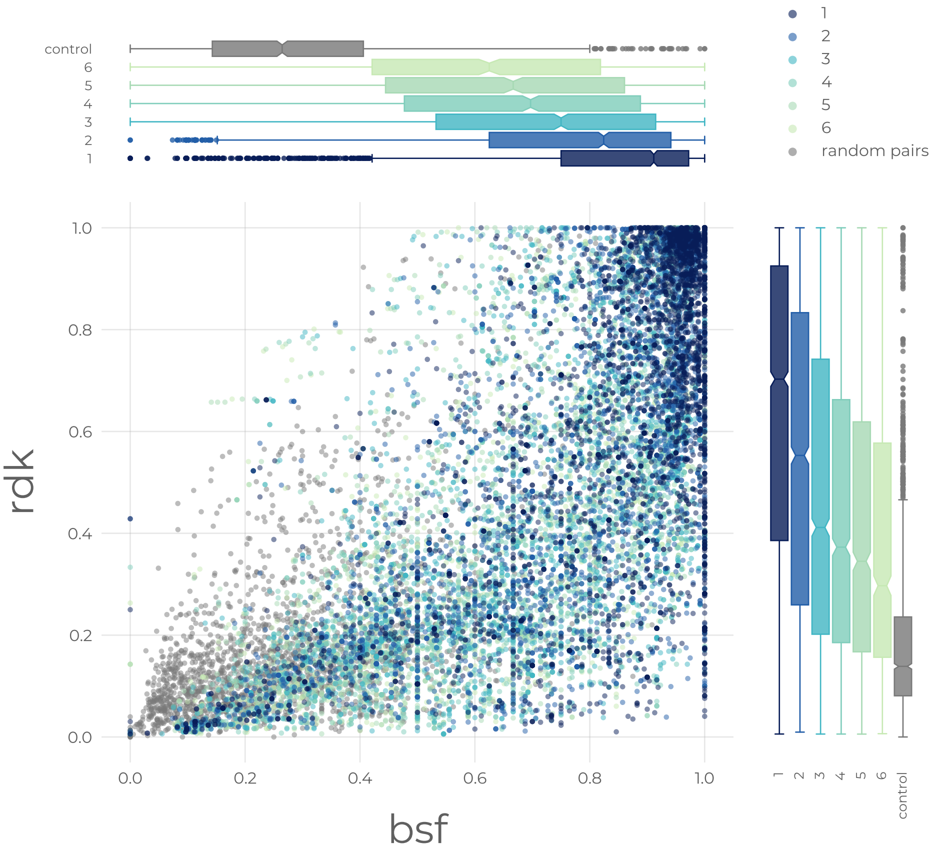

Fig. A21: Tanimoto similarity for reaction pairs: RDKit fingerprint vs Biosynfoni. These scatterplots show the individual Tanimoto similarity values of $(x, x+n)$ compound pairs from one biosynthetic reaction chain, where n is the number of biosynthetic reactions the first compound (x) goes through to become the second $(x+n)$. The increasingly lighter colours represent the value of n increasing, and compounds being increasingly distant in the reaction chain. Grey coloured dots represent random compound pairs. The boxplots on the right and the top show the distribution of the dots per n.

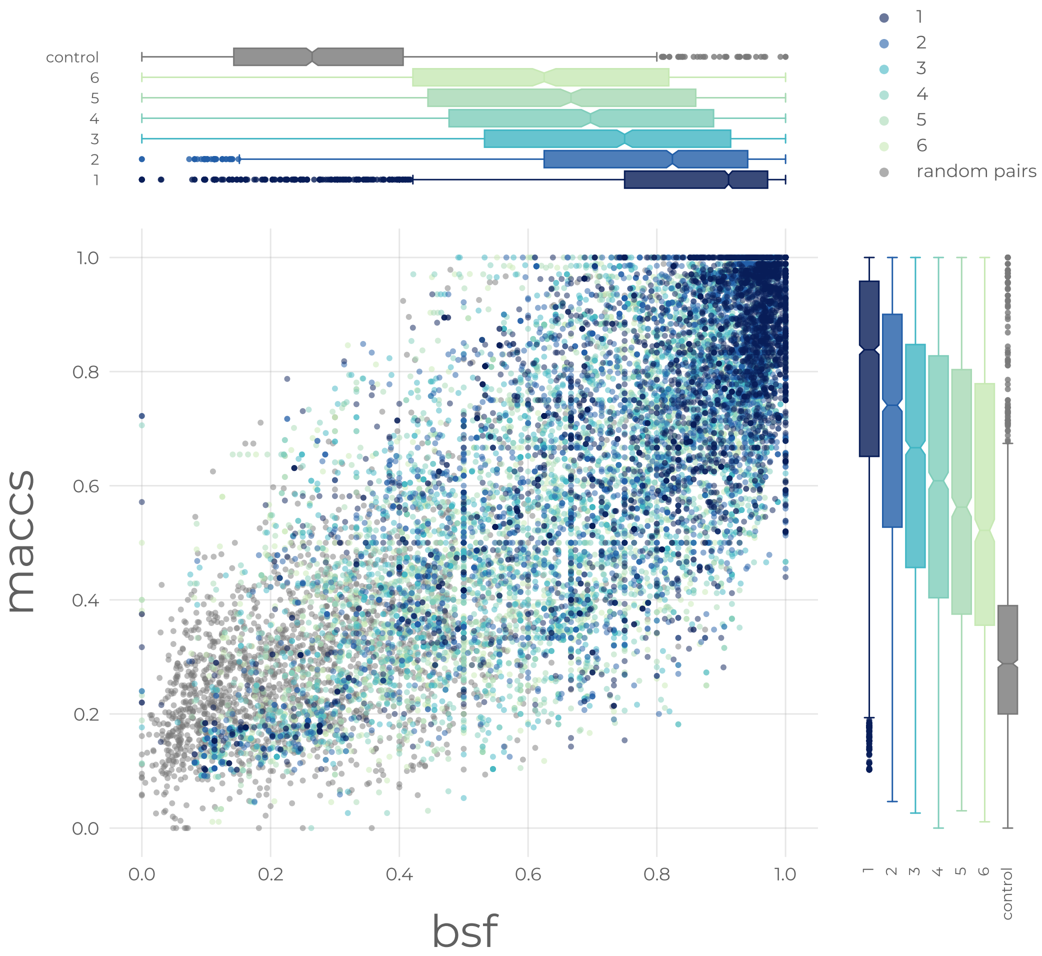

Fig. A22: Tanimoto similarity for reaction pairs: MACCS vs Biosynfoni

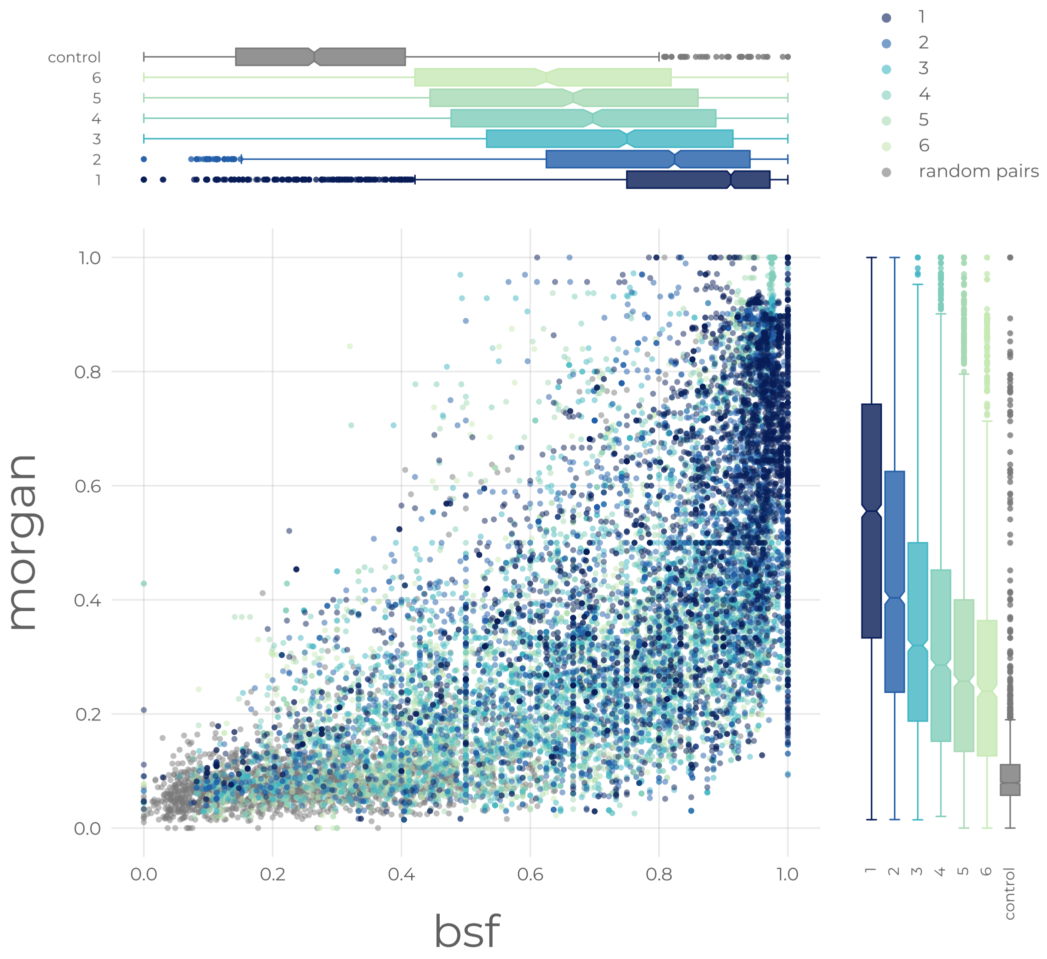

Fig. A23: Tanimoto similarity for reaction pairs: Morgan fingerprint vs Biosynfoni

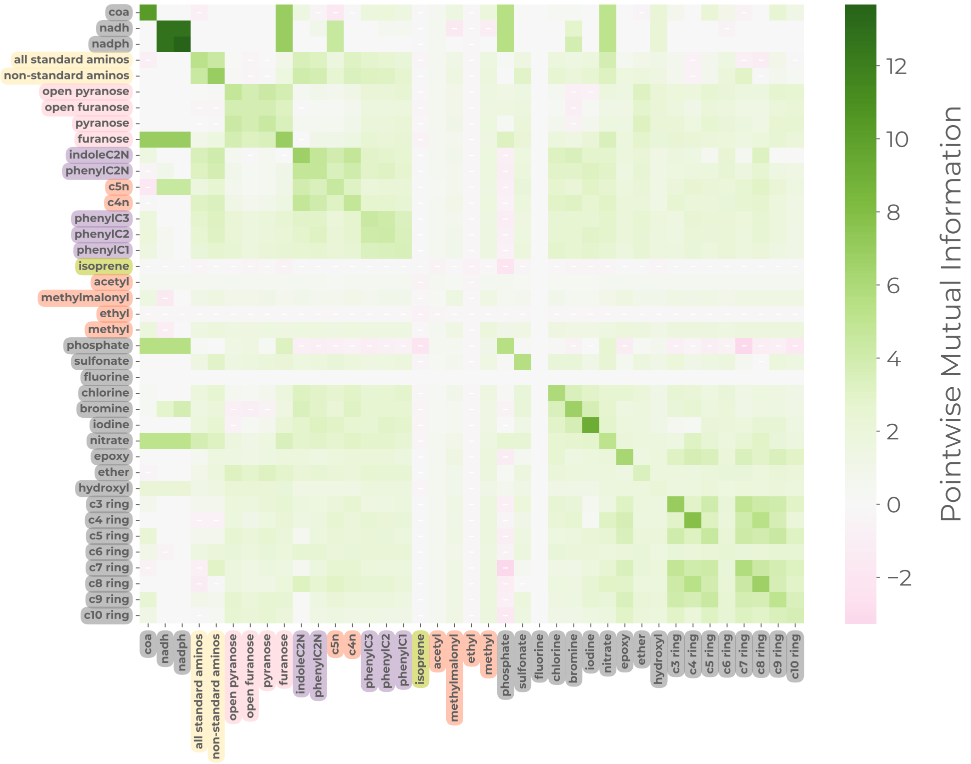

Fig. A24: Pointwise Mutual Information (PMI) plot of Biosynfoni with full substructure tags. Green colours correspond to increased PMI scores (i.e. co-occurring of substructures), pink to negative PMI scores.

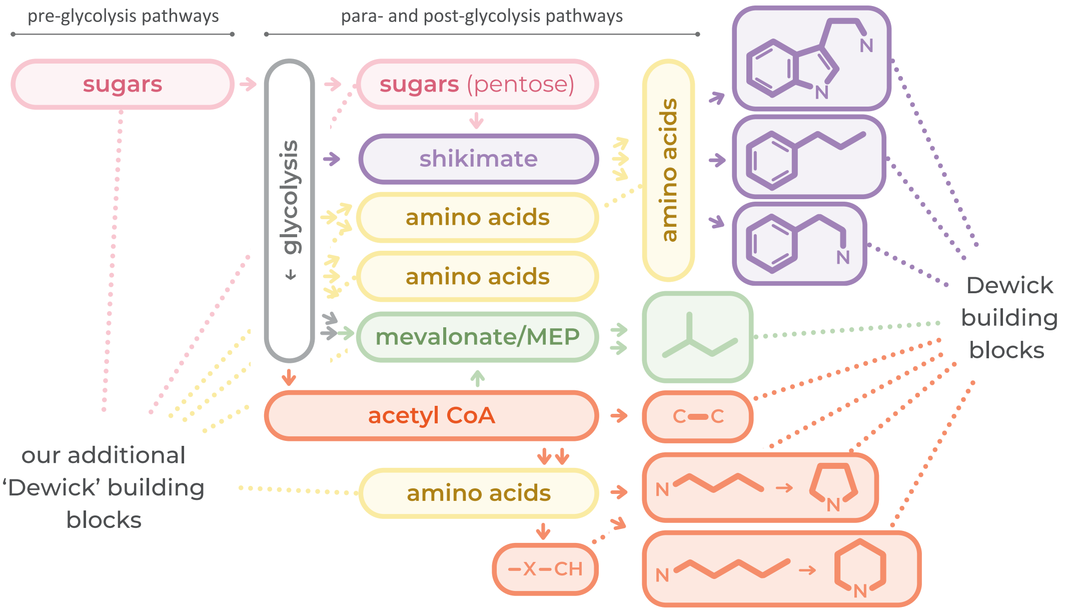

Fig. A25: The core building blocks on which Biosynfoni is based. The building blocks are based on biosynthetic logic as set out by Dewick (Dewick, 2009). 'Additional building blocks' refers to the amino acids and sugar substructures that do appear in the biosynthetic logic but not as foundational blocks (see more in section 4)

.

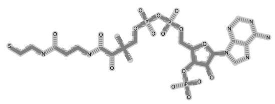

coenzyme a

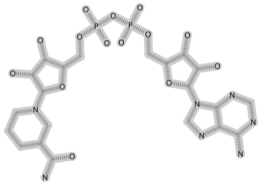

nadh

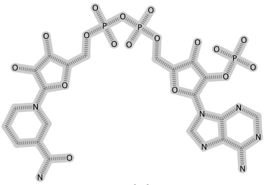

nadph

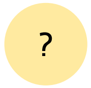

standard amino acids

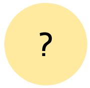

non-standard amino acids

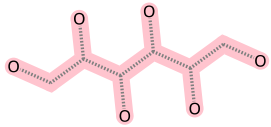

open pyranose

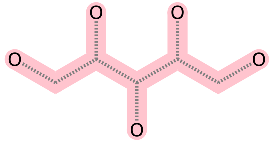

open furanose

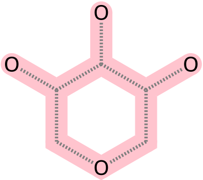

pyranose

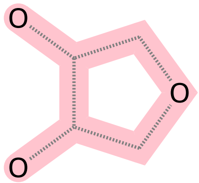

furanose

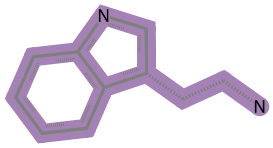

indoleC2N

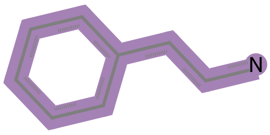

phenylC2N

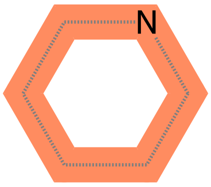

C5N

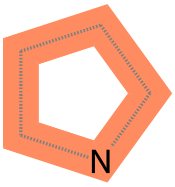

C4N

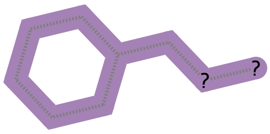

phenylC3

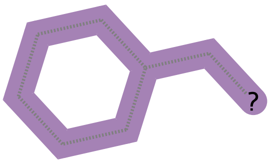

phenylC2

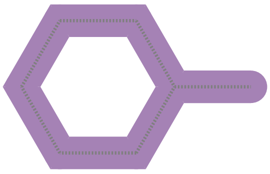

phenylC1

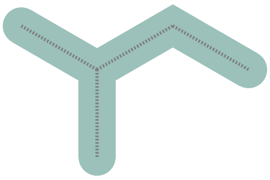

isoprene

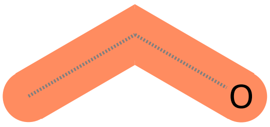

acetyl

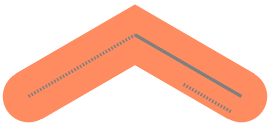

methylmalonyl

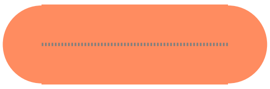

ethyl

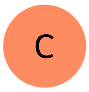

methyl

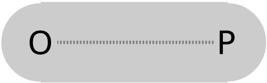

phosphate

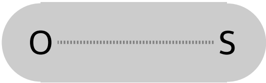

sulfonate

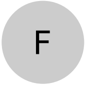

fluorine

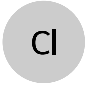

chlorine

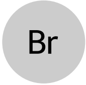

bromine

iodine

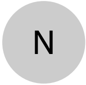

nitrate

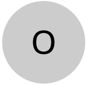

epoxy

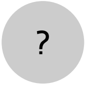

ether

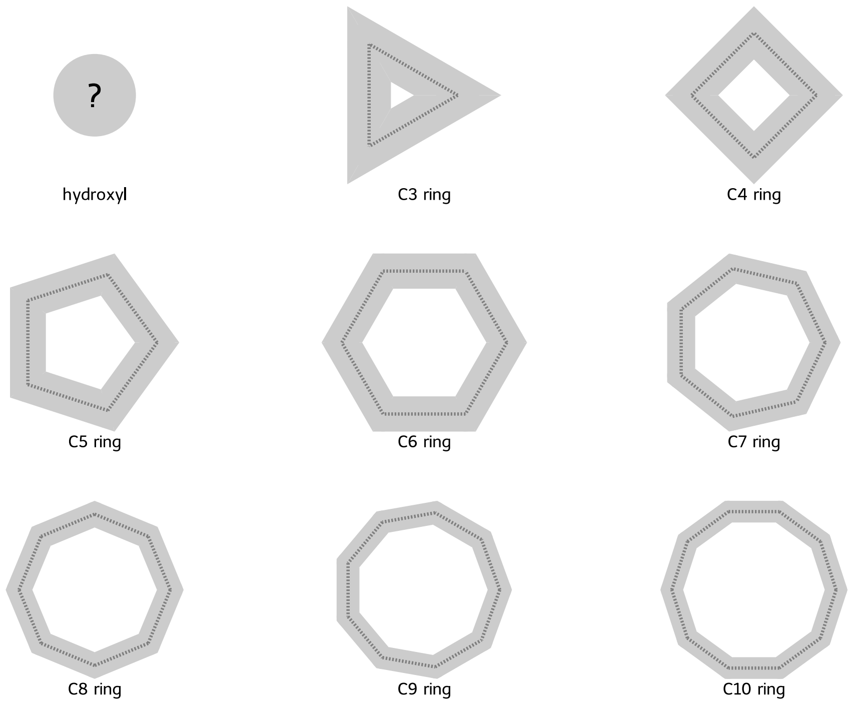

Fig. A26: SMARTS visualisations of all substructure keys used by Biosynfoni. Note that SMARTS visualisations sometimes display '?' if atom SMARTS definitions are more extensively defined (e.g. an atom that is either an oxygen or a nitrogen, or when specifying the substructure's environment). For the small two '?"s (ether and hydroxyl), a visualisation of the description is shown in the following two figures (Figure A27, Figure A28). The amino acid definitions are too large to visualise. The full SMARTS definitions can be accessed in-code through the Biosynfoni.subkeys module.

[O;D2;!h;!$(*C=O);X2;!R;!$(*P);!$(*S)]

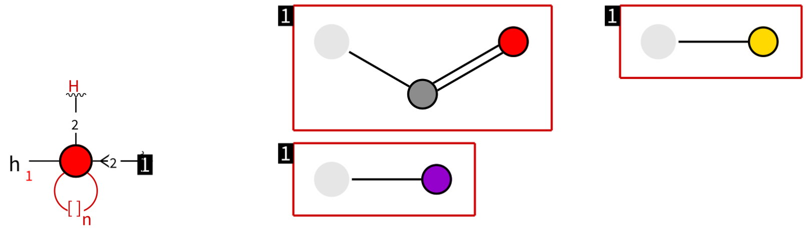

Picture created by the SMARTSviewer [https://smarts.plus/].
Copyright: ZBH - Center for Bioinformatics Hamburg.

LEGEND

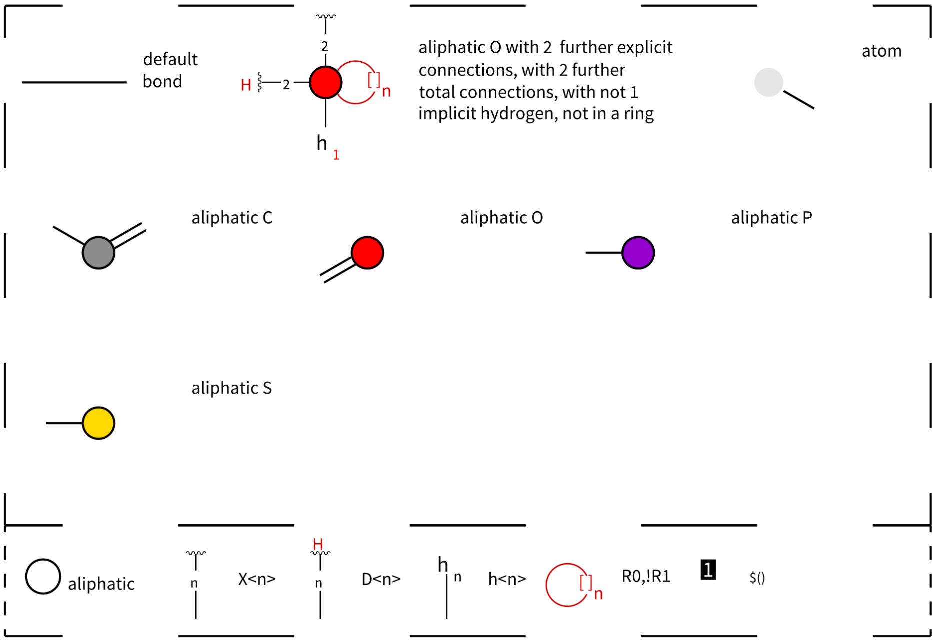

Fig. A27: SMARTS visualisation for the ether substructure

[#8;D1;h,!v2;$(*[#6,#7]);!$(*C~O);!$(P);!$(S)]

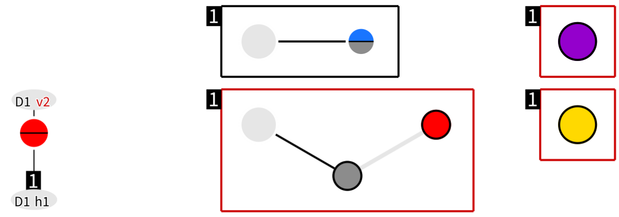

Picture created by the SMARTSviewer [https://smarts.plus/].
Copyright: ZBH - Center for Bioinformatics Hamburg.

LEGEND

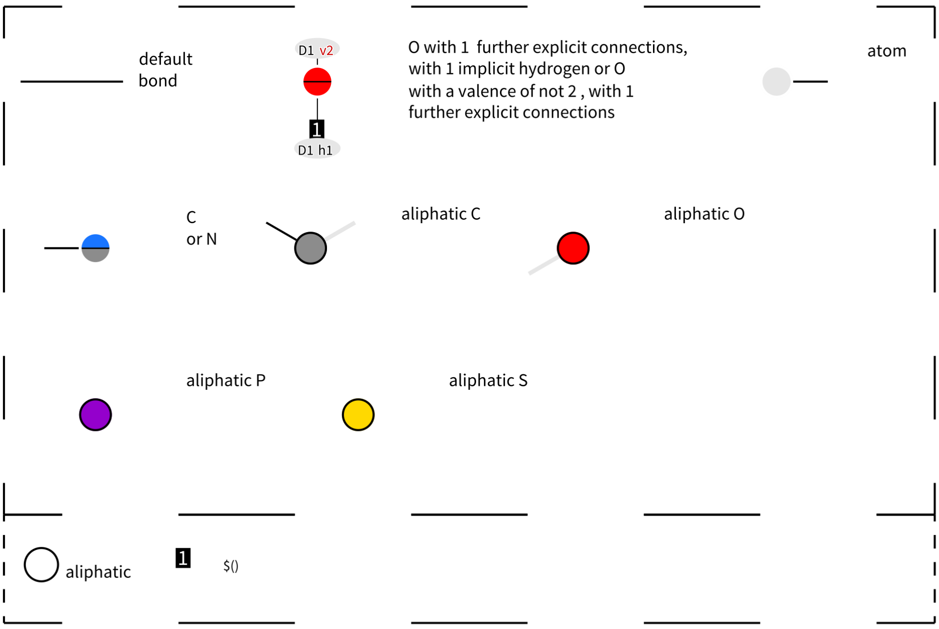

Fig. A28: SMARTS visualisation for the hydroxyl substructure

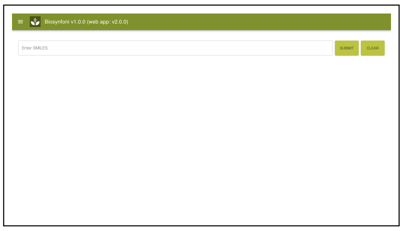

(a) Step 1: Start page of Biosynfoni prompts the user to submit a SMILES string.

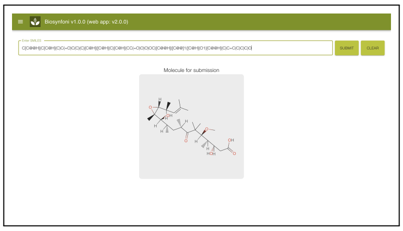

(b) Step 2: Users can easily write or copy in the SMILES string of their liking. Valid SMILES will be drawn by the Smiles-Drawer element (Probst and Reymond, 2018). When happy with the input, users can press the "Submit" button. This will send the input SMILES to the backend where it will be processed and used as input for the biosynthetic class prediction.

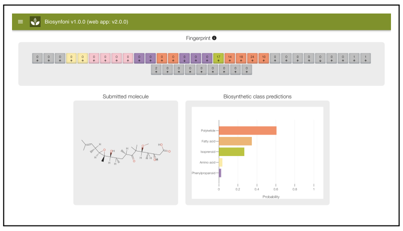

(c) Step 3: When the prediction results are ready, these will be shown on the same screen below the SMILES input field. The results exist of three sections: the Biosynfoni fingerprint generated for the input SMILES (i.e., Fingerprint section), the drawn submitted compound (i.e., Submitted molecule section), and the biosynthetic class predictions (i.e., Biosynthetic class predictions section).

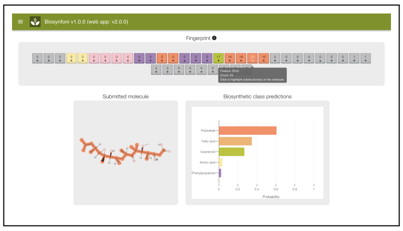

(d) Step 4: Users can save the class predictions using the plot's interface. Users are also able to get insight into how the Biosynfoni fingerprint was constructed by selecting individual features. The selected features will be highlighted in the drawn molecule in the Submitted molecule section. Additionally, the upwards or downwards-facing arrow alongside each feature indicates if the feature contributed positively or negatively to the top predicted class.

Fig. A29: Overview of the Biosynfoni web application with step-by-step interface interactions. Biosynfoni can be visited at moltools.bioinformatics.nl/biosynfoni. The code for the Biosynfoni web application is part of MolTools, which source code can be found at https://github.com/moltools/moltools. The version of the Biosynfoni web application shown here is part of MolTools v2.0.0. The backend of MolTools interacts with the Biosyfoni package for the biosynthetic class predictions. The model used for the biosynthetic class predictions in MolTools v2.0.0 is the bsf_model model, which can be downloaded from Zenodo at https://zenodo.org/records/14791239. Determining if a feature contributed positively or negatively to the top predicted class was performed by the MolTools backend with TreeExplainer implemented in shap (Lundberg et al, 2020).

Table A1: ChEBI class sizes

Class	Number of compounds
alkaloid	909
amino acid	2543
carbohydrate	2679
fatty acid	10190
isoprenoid	4127
phenylpropanoid	1686
polyketide	475

Table A2: Dependency versions

Name	Version
Python	3.13.1
RDKit	2024.9.4
Numpy	2.1.3
Pandas	2.2.3
tqdm	4.67.1
setuptools	75.8.0
Matplotlib	3.10.0
Scikit-learn	1.6.1
Scipy	1.15.1
umap-learn	0.5.7
requests	2.32.3
seaborn	0.13.2
jupyter	1.1.1

Table A3: Database versions

	version	date downloaded	downloaded from
**COCONUT**	2 (2024-09)	2024-10-02	https://coconut.naturalproducts.net/download
**MetaCyc**	27.1	2023-09-22	requested personal link (free usage for academic purposes)
**ChEBI (.owl)**	2023-10-27	2023-11-28	https://ftp.ebi.ac.uk/pub/databases/chebi/
**ChEBI 3star (.sdf)**	2023-10-27	2023-12-06	https://ftp.ebi.ac.uk/pub/databases/chebi/
**ZINC**	2	2023-09-06	reference data of Sorokina et al. (2019) https://zenodo.org/record/2652372[1]

[1]due to difficulties with main ZINC site downloads
